# “Recognition of heart attack symptoms and treatment-seeking behaviors: a multi-center survey in Tehran, Iran”

**DOI:** 10.1186/s12889-023-15826-1

**Published:** 2023-05-12

**Authors:** Elnaz Shahmohamadi, Mojtaba Sedaghat, Arash Rahmani, Farnoosh Larti, Babak Geraiely

**Affiliations:** 1 Department of Cardiology, Imam Khomeini Hospital Complex, Tehran University of Medical Sciences, Tehran, 1419733141 Iran; 2 Department of Community Medicine, Faculty of Medicine, Tehran University of Medical Science, Tehran, Iran

**Keywords:** Myocardial Infarction, Awareness, Questionnaire development, Symptoms, Iran

## Abstract

**Background:**

In acute myocardial infarction (AMI), timely recognition of symptoms and early hospital presentation positively affect patient morbidity and mortality. Due to the high burden of ischemic heart disease in Iran, this study aimed to identify factors affecting the level of knowledge, responses at the time of AMI onset, and sources of health information among the Iranian population.

**Method:**

This cross-sectional study was conducted in three tertiary hospitals in Tehran, Iran. An expert-validated questionnaire was used to obtain data. A total of 400 individuals were enrolled.

**Result:**

Among the respondents, 285 people(71.3%) considered “chest pain or discomfort,” and 251 (62.7%) regarded “pain or discomfort in the arm or shoulder” as MI symptoms. Approximately 288 (72.0%) respondents had poor knowledge of the AMI symptoms. Knowledge of symptoms was higher among those with higher levels of education, those with medical-associated jobs, and those who resided in the capital areas. Major risk factors identified by the participants were: anxiety (340)(85.0%), obesity (327)(81.8%), an unhealthy diet (325)(81.3%), and the presence of high LDL levels (258)(64.5%) and Diabetes Mellitus (164)(41.0%) were less appreciated. Calling an ambulance (286)(71.5%) was the most common treatment-seeking behavior in the case of a suspected heart attack.

**Conclusion:**

It is vital to educate the general population about AMI symptoms, particularly those with comorbidities at the greatest risk for an AMI episode.

## Introduction

Ischemic heart disease (IHD) is the leading cause of death globally, with approximately 9.1 million deaths in 2019 [1]. People who suffer an acute myocardial infarction (AMI) can experience severe morbidity and are at an increased risk for subsequent AMIs [2]. According to the Global Burden of Disease (GBD) study, ischemic heart disease is the top-ranked cause of disability-adjusted life years (DALYs) in people over the age of 50 [3]. In Iran, ischemic heart disease accounted for 29.9 percent of all deaths in 2019[3]. In addition, the burden of IHD is expected to increase as the population ages.

The most common presenting symptoms of AMI are chest, shoulder, or arm pain, dyspnea, unexplained weakness, nausea, or a combination of these [4]. In AMI, timely recognition of symptoms, early presentation to the hospital, and medical treatment have an unconditional impact on patient morbidity and mortality. The idiom "time is muscle" underscores the importance of saving time and initiating therapy immediately[5]. However, prehospital delay of MI is common worldwide[6, 7]. Patients with AMI may delay seeking treatment due to denial or attribution of symptoms to other etiologies[8]. According to a study conducted in Iran, unawareness of IHD symptoms seems to be the most common reason for prehospital delay[9].

In acute MI, patients' awareness of symptoms and ways of expressing them were often related to information from multimedia or other sources such as their relatives, friends, or healthcare providers, [10]. A study found that more than half of patients diagnosed with AMI learned about its symptoms from healthcare providers and the mass media[11]. Moreover, reliance on relatives as the primary source of information has been associated with a widening gap between experienced and expected symptoms of myocardial infarction [10].

As far as we know, this is the first study to develop and evaluate a questionnaire to determine awareness of AMI symptoms and treatment-seeking behaviors in a sample of Iranian adults. We aimed to identify factors affecting the level of knowledge, responses at the time of AMI onset, and sources of health information in this developing country.

## Methods

### Study population

The study population was selected from patients with cardiac or medical (non-cardiac) diseases or their relatives. The study was conducted from January to July 2022 in three general tertiary hospitals in Tehran, Iran: Imam Khomeini, Shariati, and Amir Alam. Four hundred consecutive participants were selected from the emergency department, cardiology, internal medicine wards, and outpatient clinics. A cluster-stratified randomized sampling approach was used, where the probability of each individual being selected is equal and proportional to the number of individuals referring to each institution. Individuals between the ages of 25 and 64 were eligible for inclusion, while all healthcare professionals affiliated with academic institutions were excluded. All participants were informed that their confidentiality would be protected and that the data collected would be used for research purposes only.

### Questionnaire development

The questionnaire has been modified from several international studies involving different races [12–17]. Details of the study methodology and questionnaire validation will be published shortly. In summary, it was assessed in a pilot study with 40 participants who met the criteria for enrollment in this study. As proposed by Rattray and Jones, [18], a sample size of fewer than 100 individuals are sufficient to design and develop questionnaires. After conducting the pilot study, some minor adjustments were made. After sending the draft questionnaire to five experts in the field for face and content validity evaluations, a final version was created. After integrating the experts’ opinions, the necessary modifications were made. The final questionnaire contained fours parts as follows:

The first part of the questionnaire elicited the sociodemographic information of the respondents, including their sex, age, educational level, employment status, and area of residence, as well as their medical history information, including the history of hypertension, diabetes, hyperlipidemia, IHD, smoking, opium use, and MI in the patient or the immediate relatives).

The second part of the questionnaire assessed participants’ awareness of MI symptoms. Patients were asked, "Which of the following do you believe is a symptom of an acute heart attack?" and were given a list of symptoms, including ‘chest pain, discomfort,’ ‘Pain or discomfort in the jaw, neck, or back, ‘Feeling weak, light-headed, or faint,’ ‘pain or discomfort in the arm/shoulder,’ ‘Dyspnea,’’ Diaphoresis,’’ Nausea or vomiting, and’Abdominal pain/discomfort as well as two trap answers (blurred vision and headache) to determine if the respondent answered “yes” to all the symptoms in the closed-ended question series. “Excellent knowledge of MI symptoms” was defined as correctly identifying at least five out of eight correct symptoms of MI. Choosing trap answers didn’t result in a negative score. Although respondents who answered 'yes' to all questions would be excluded from the analysis, we found no instances of this in any questionnaires.

In the third part, participants were asked, “Assume you had an acute heart problem, what would you do first?” and were given a list of behaviors, including going to the emergency room, calling an ambulance, resting and taking medicine, going to the hospital, contacting a family doctor, calling family members, waiting, and other things., Respondents were also presented with two imaginary MI scenarios:

- Scenario 1: You have pain in your arm, shoulder, and abdomen, accompanied by a feeling of indigestion, lasting thirty minutes during the day or night
- Scenario 2: You have intense, severe chest discomfort throughout the day or night.

These two case scenarios were given to evaluate the effect of symptom type and chest pain intensity on their expected treatment-seeking behavior. In scenario 1, pain in the arm, shoulder, and abdomen, was mentioned along with a feeling of indigestion, was mentioned. In scenario 2, the chest was explicitly mentioned as a cue to participants about the cardiac origin and intensity of the pain to determine whether it would be critical to the decision to seek treatment. The expected treatment-seeking behavior of the participants in each scenario was assessed using first-responder questions. “Appropriate treatment-seeking behavior*”* was determined by the correct responses of "going to the emergency room " and "calling an ambulance" to both scenarios.

In the final section, respondents were asked about the source of their health-related information. During the completion of the questionnaires, the researchers were available to provide clarification and to verify that all items had been answered.

### Definition of variables

The study's independent variables included demographic characteristics such as sex, age, education, area of residency, and race. Patient medical status was described as “Patient with CVD, outpatient,” “Patient with CVD, inpatient,” “Patient with non-CVD, outpatient,” “Patient with non-CVD, inpatient,” “Immediate relative of patients with CVD,” and “Immediate relative of patients with CVD.” Cardiovascular disease (CVD) includes ischemic heart disease, cerebrovascular disease, valvular heart disease, and cardiomyopathies. Regular opium user was defined as daily opiate use. Exercise is described as physical activity that results in increased heart rate, ventilation, and sweating.

### Statistical analysis

We used IBM SPSS Statistics for Windows version 25 (IBM Corp., Armonk, NY, USA) for data analysis. Descriptive analyses were used to describe the variables. Chi-squared tests were used to compare the awareness of each symptom and knowledge of AMI symptoms based on socio-demographic characteristics, including age, gender, area of residence, education level, jobs, as well as clinical characteristics, including a history of MI and comorbidities such as hypertension, diabetes, IHD, and dyslipidemia.

## Results

### Baseline characteristics

Table 1 shows the sociodemographic and clinical characteristics of the study population. A total of 400 participants were included in the final analysis. The mean age of the participants was 42.0 years (± 12.6 SD). Of the respondents, 206 (51.5%) were female, 283 (70.8%) were married, and the majority, 380 (95%), were literate. Among the study population, 34 people (8.5%) were unemployed, and 20 (5%) had a job related to medical care. 67(16.8%) of the participants were current smokers, 4(1.0%) were regular opium users, and 261(65.3%) had insufficient physical activity. Regarding the medical history of the respondents, the percentages for diabetes, hypertension, IHD, and hyperlipidemia (HLP) were 44(11.0%), 62(15.5%), 47(11.8%), and 60(15%), respectively. Approximately 3% (13 subjects) of the study population reported a previous history of MI in themselves.

**Table 1:** The sociodemographic and clinical characteristics of the study population

Variable	Value
Age	42.0 ± 12.6
Sex	
Male	194 (48.5)
Female	206 (51.5)
Marital status	
Married	283 (70.8)
Education	
Illiterate	20 (5.0)
Undergraduate	90 (22.4)
Diploma	165 (41.3)
Bachelor or higher	125 (31.3)
Employment status	
Unemployed	34 (8.5)
Retired	31 (7.8)
Housewife	103 (25.4)
Student	13 (3.3)
Self-employed	135 (33.8)
Employed	56 (14.0)
Medical associated	20 (5.0)
Other	8 (2.0)
Race	
Iranian	385 (96.3)
Other	15 (3.7)
Area of Residence	
Urban- Capital area	198 (49.5)
Urban- Provincial area^a^	181 (45.3)
Rural	21 (5.2)
History of MI	
Respondent	13 (3.3)
Immediate family member	139 (34.7)
None	248 (62.0)
Medical status of the respondents	
Patient with CVD, outpatient	20 (5.0)
Patient with CVD, inpatient	23 (5.8)
Patient with non-CVD, outpatient	64 (16.0)
Patient with non-CVD, inpatient	87 (21.8)
Immediate relative of patients with CVD	44 (11.0)
Immediate relative of patients with non-CVD	135 (33.8)
Other	27 (6.8)
Smoking	
Current smoker	67 (16.7)
Ex-smoker	40 (10.0)
Never	293 (73.3)
Opium	
Occasional user	11 (2.8)
Regular user	4 (1.0)
Ex-user	26 (6.5)
Never	359 (89.7)
30 Minutes exercise/week	
0–2 times	261 (65.2)
3–5 times	74 (18.5)
More than 5 times	65 (16.3)
Comorbidities	
HTN	62 (15.5)
DM	44 (11.0)
HLP	60 (15.0)
IHD	47 (11.8)
None	187 (46.7)

^a^Other districts of province

*Abbreviations*: *MI* Myocardial Infarction, *CVD* Cardiovascular diseases, *HTN* Hypertension, *DM* Diabetes Mellitus, *HLP* Hyperlipidemia, *IHD* Ischemic Heart Disease

Data is presented as mean ± standard deviation for quantitative variables, and n (%) for categorical variables

### Awareness of myocardial infarction symptoms

Among the respondents, 285(71.3%) considered “chest pain or discomfort,” and 251(62.7%) considered “pain or discomfort in the arm or shoulder” as MI symptoms. Fewer participants reported that dyspnea, 207(51.7%), and diaphoresis, 150(37.5%), were symptoms. The least commonly identified symptom was “abdominal pain or discomfort” 56(14.0%). Moreover, 25.0% (100 subjects) and 19.0% (76 subjects) of respondents answered: “yes” to the trap questions “blurred vision” and “headache,” respectively. There were few differences between the sexes in recognition of MI symptoms. However, men were significantly less likely to identify “Pain or discomfort in the jaw, neck, or back” as a symptom (*p*-value = 0.003).

There were also differences in the identification of symptoms based on comorbidities. Respondents with an immediate family history of MI were significantly more likely to recognize diaphoresis and nausea or vomiting as MI symptoms (*p*-values = 0.001, 0.007, respectively). There was no significant difference between the respondents with and without comorbidities in recognizing MI symptoms. Awareness of each symptom of MI according to sociodemographic and clinical characteristics is shown in Table 2.

**Table 2:** Awareness of acute myocardial infarction symptoms according to the sociodemographic and clinical characteristics of participants

acute myocardial infarction symptoms		Pain or discomfort in the jaw, neck, or back	Feeling weak, light-headed, or faint	chest pain, discomfort	blurred vision	pain or discomfort in the arm/shoulder	Dyspnea	Headache	Diaphoresis	Nausea/ vomiting	Abdominal pain/ discomfort
**Characteristics**											
Total		38%	30.8%	71.3%	25.0%	62.7%	51.7%	19%	37.5%	27.3	14.0
Sex	Males	30.4%	27.8%	67.5%		59.3%	45.4%		30.9%	22.2	14.9
	Females	45.1%	33.5%	74.8%		66.0%	57.8%		43.7%	32.0	13.1
	***p*-value**	**0.003**	**0.234**	**0.122**		**0.179**	**0.016**		**0.010**	**0.033**	**0.666**
Age	***p*-value**	**0.499**	**0.179**	**0.897**		**0.464**	**0.205**		**0.270**	**0.533**	**0.402**
Education	Illiterate	15%	25.0%	25.0%		40.0%	35.0%		20.0%	20.0	20.0
	Undergraduate	21.1%	17.8%	56.7%		51.1%	41.1%		16.7%	18.9	11.1
	Diploma	40.6%	34.5%	79.4%		67.3%	53.9%		41.8	30.9	18.2
	Bachelor or higher	50.4%	36.0%	78.4%		68.8%	59.2%		49.6	29.6	9.6
	***p*-value**	**0.000**	**0.017**	**0.000**		**0.005**	**0.024**		**0.000**	**0.160**	**0.131**
Job	Medical associate	65%	40.0%	80.0%		85.0%	75.0%		50.0	50.0	25.0
	Non-medical	36.6%	30.3%	70.8%		61.6%	50.5%		36.8	26.1	13.4
	***p*-value**	**0.016**	**0.456**	**0.456**		**0.035**	**0.039**		**0.245**	**0.035**	**0.177**
Nationality	Iranian	39.5%	31.2%	72.7%		64.2%	52.5%		38.4	27.5	13.8
	Others	0%	20.0%	33.3%		26.7%	33.3%		13.3	20.0	20.0
	***p*-value**	**0.001**	**0.569**	**0.002**		**0.005**	**0.190**		**0.058**	**0.768**	**0.451**
1Living place	Urban-Capital area	44.9%	32.8%	78.3%		69.7%	54.0%		44.9	31.3	16.2
	Urban- Provincial areas^a^	32.0%	28.7%	64.6%		53.6%	49.2%		30.4	23.8	11.6
	Rural area	23.8%	28.6%	61.9%		76.2%	52.4%		28.6	19.0	14.3
	***p*-value**	**0.014**	**0.672**	**0.008**		**0.002**	**0.637**		**0.010**	**0.176**	**0.442**
History of MI	Respondent	15.4%	39.8%	76.9%		61.5%	46.2%		23.1	15.4	15.4
	Immediate relative	43.9%	33.1%	77.0%		71.9%	61.2%		49.6	36.7	15.1
	None	35.9%	29.4%	67.7%		57.7%	46.8%		31.5	22.8	13.3
	***p*-value**	**0.069**	**0.756**	**0.141**		**0.020**	**0.023**		**0.001**	**0.007**	**0.877**
Medical status	Patient with CVD	27.9%	37.2%	83.7%		67.4%	48.8%		37.2	25.6	18.6
	Patient with non-CVD	33.1%	24.5%	63.6%		57.6%	46.4%		33.1	23.2	12.6
	Immediate relative of patients with CVD	34.1%	47.7%	77.3%		65.9%	59.1%		43.2	29.5	18.2
	Immediate relative of patients with non-CVD	50.4%	29.6%	79.3%		70.4%	57.0%		43.7	34.1	14.1
	None	25.9%	33.3%	44.4%		40.7%	48.1%		22.2	14.8	7.4
	***P*-value**	**0.007**	**0.045**	**0.000**		**0.024**	**0.342**		**0.150**	**0.152**	**0.623**
HTN	Yes	46.8%	35.5%	74.2%		67.7%	58.1%		38.7%	32.3%	14.5%
	No	36.4%	29.9%	70.7%		61.8%	50.6%		37.3%	26.3%	13.9%
	***P*-value**	**0.154**	**0.374**	**0.649**		**0.396**	**0.333**		**0.887**	**0.353**	**0.844**
DM	Yes	38.6%	31.8%	65.9%		54.5%	52.3%		38.6%	29.5%	13.6%
	No	37.9%	30.6%	71.9%		63.8%	51.7%		37.4%	27.0%	14.0%
	***P*-value**	**1.000**	**0.864**	**0.480**		**0.250**	**1.000**		**0.870**	**0.721**	**1.000**
HLP	Yes	35.0%	33.3%	66.7%		63.3%	50.0%		41.7%	35.0%	15.0%
	No	38.5%	30.3%	72.1%		62.6%	52.1%		36.8%	25.9%	13.8%
	***P*-value**	**0.666**	**0.651**	**0.439**		**1.000**	**0.781**		**0.473**	**0.158**	**0.840**
IHD	Yes	29.8%	27.7%	78.7%		68.1%	59.6%		44.7%	29.8%	14.9%
	No	39.1%	31.2%	70.3%		62.0%	50.7%		36.5%	26.9%	13.9%
	***P*-value**	**0.263**	**0.737**	**0.303**		**0.521**	**0.279**		**0.336**	**0.728**	**0.824**

^a^Other districts of province

*Abbreviations*: *MI* Myocardial Infarction, *CVD* Cardiovascular diseases, *HTN* Hypertension, *DM* Diabetes Mellitus, *HLP* Hyperlipidemia, *IHD* Ischemic Heart Disease

Approximately 72.0% of the respondents (288 subjects) had poor knowledge of AMI symptoms. Women better understood AMI symptoms than men (35.0% vs. 20.6%, *p*-value: 0.002). Knowledge of symptoms was higher among those with a higher level of education, those with medical-associated jobs, and those living in the capital areas than among those with a lower level of education, those with non-medical-associated jobs, and those living in provincial or rural areas, respectively. Additionally, respondents with a family history of MI had better knowledge than those with a personal history of MI (Table 3).

**Table 3:** Knowledge of acute myocardial infarction symptoms according to sociodemographic and clinical characteristics of participants

Characteristics		Excellent Knowledge	Poor knowledge
Age	*p*-value	112 (28.0)	288 (72.0)
		**0.359**	
Sex	Males	40 (20.6)	154 (79.4)
	Females	72 (35.0)	134 (65.0)
	***p*-value**	**0.002**	
Education	Illiterate	4 (20.0)	16 (80.0)
	Undergraduate	15 (16.7)	75 (83.3)
	Diploma	50 (30.3)	115 (69.7)
	Bachelor or higher	43 (34.4)	82 (65.6)
	***p*-value**	**0.025**	
Job	Medical associated	10 (50.0)	10 (50.0)
	Non-medical	102 (26.8)	278 (73.2)
	***p*-value**	**0.038**	
Nationality	Iranian	110 (28.6)	275 (71.4)
	Others	2 (13.3)	13 (86.7)
	***p*-value**	**0.252**	
Area of residence	Urban-Capital areas	67 (33.8)	131 (66.2)
	Urban-Provincial areas^a^	40 (22.1)	141 (77.9)
	Rural areas	5 (23.8)	16 (76.2)
	***p*-value**	**0.036**	
History of MI	Respondent	1 (7.7)	12 (92.3)
	Immediate family	57 (41.0)	82 (59.0)
	none	54 (21.8)	194 (78.2)
	***p*-value**	**0.000**	
Medical Status	Patient with CVD (outpatient/inpatient)	8 (18.6)	35 (81.4)
	Patient with non-CVD (outpatient/inpatient)	37 (24.5)	114 (75.5)
	Immediate relative of patients with CVD	18 (40.9)	26 (59.1)
	Immediate relative of patients with non-CVD	46 (34.1)	89 (65.9)
	None	3 (11.1)	24 (88.9)
	***P*-value**	**0.013**	
HTN	Yes	18 (29.0%)	44 (71.0%)
	No	94 (27.8%)	244 (72.2%)
	***P*-value**	**0.878**	
DM	Yes	12 (27.3%)	32 (72.7%)
	No	100 (28.1%)	256 (71.9%)
	***P*-value**	**1.000**	
HLP	Yes	21 (35.0%)	39 (65.0%)
	No	91 (26.8%)	249 (73.2%)
	***P*-value**	**0.213**	
IHD	Yes	13 (27.7%)	34 (72.3%)
	No	99 (28.0%)	254 (72.0%)
	***P*-value**	**1.000**	

^a^Other districts of province

*Abbreviations*: *MI*:Myocardial Infarction, *CVD* Cardiovascular diseases, *HTN* Hypertension, *DM* Diabetes Mellitus, *HLP* Hyperlipidemia, *IHD* Ischemic Heart Disease

Major risk factors identified by participants included: anxiety, 340(85.0%), obesity, 327(81.8%), unhealthy diet, 325(81.3%), and tobacco/hookah smoking, 322(80.5%). Of the respondents, 45.5% (182 subjects) were unsure whether diabetes was a risk factor for myocardial infarction. Only 3.5 percent of the participants(14 subjects) didn't identify any risk factors for MI, while 23.8 percent (95 subjects) were able to identify all risk factors for MI (Table 4).

**Table 4:** Awareness of role of risk factors associated with myocardial infarction

**Risk factors**		Value
Tobacco/ Hookah	Yes	322 (80.5)
	No	6 (15)
	I don’t know	72 (18.0)
Unhealthy diet (with high levels of Salt, statured fat, and cholesterol)	Yes	325 (81.3)
	No	8 (2.0)
	I don’t know	67 (16.8)
Insufficient physical activity	Yes	301 (75.3)
	No	28 (7.0)
	I don’t know	71 (17.8)
Obesity	Yes	327 (81.8)
	No	19 (4.8)
	I don’t know	54 (13.5)
Anxiety	Yes	340 (85.0)
	No	10 (2.5)
	I don’t know	50 (12.5)
Genetics and family history of CVD	Yes	277 (69.3)
	No	21 (5.3)
	I don’t know	102 (25.5)
High serum level of LDL	Yes	258 (64.5)
	No	18 (4.5)
	I don’t know	124 (31.0)
Diabetes	Yes	164 (41.0)
	No	54 (13.5)
	I don’t know	182 (45.5)
HTN	Yes	311 (78.8)
	No	10 (2.5)
	I don’t know	79 (19.8)
Alcohol	Yes	235 (58.8)
	No	37 (9.3)
	I don’t know	128 (32.0)
Number of RFs identified by each respondent	0	14 (3.5)
	1	9 (2.3)
	2	6 (1.5)
	3	13 (3.3)
	4	23 (5.8)
	5	33 (8.3)
	6	46 (11.5)
	7	36 (9.0)
	8	60 (15.0)
	9	65 (16.3)
	10	95 (23.8)

Data expressed as number (percent)

*Abbreviations*: *CVD* Cardiovascular diseases, *HTN* Hypertension, *LDL* Low-density Lipoprotein, *RF* Risk factor

### Treatment-seeking behaviors

As shown in Table 5, calling an ambulance, 286(71.5%) was the most common treatment-seeking behavior in the case of a suspected heart attack. The proportion of respondents who opted for calling an ambulance was greater in scenario 2 (‘intense chest pain’) than in scenario 1 (“pain in arms, shoulders or abdomen”). Moreover, attending the emergency room was the most popular expected treatment-seeking behavior in Scenario 1. Appropriate treatment-seeking behavior was reported in 157 subjects (39.3%) for both scenarios. Furthermore, older age and having a history of HTN were associated with a lower likelihood of appropriate anticipated treatment-seeking behavior (Table 6).

**Table 5:** Different responses in time of heart attack and response to imaginary scenarios

Action	Heart attack	Scenario 1	Scenario 2
ER attendance	79 (19.8)	107 (26.8)	124 (31.0)
Calling an ambulance	286 (71.5)	64 (16.0)	173 (43.3)
Rest and take medicine	7 (1.8)	16 (4.0)	6 (1.5)
Hospital attendance	1 (0.3)	55 (13.8)	27 (6.8)
Call family doctor	8 (2.0)	33 (8.3)	16 (4.0)
Call family members	15 (3.8)	28 (7.0)	31 (7.8)
Wait	1 (0.3)	71 (17.8)	10 (2.5)
Other	0	5 (1.3)	2 (0.5)
Doesn’t know	3 (0.8)	21 (5.3)	11 (2.8)

Data expressed as number(percent)

**Table 6:** Factors associated with appropriate treatment-seeking behavior in hypothetical AMI scenarios

Characteristics		Appropriate treatment-seeking behavior
Age		157 (40.32)
	***p*-value**	**0.030**
Sex	Males	80 (41.2)
	Females	77 (37.4)
	***p*-value**	**0.474**
Education	Illiterate	3 (15.0)
	Undergraduate	32 (35.6)
	Diploma	65 (39.4)
	Bachelor or higher	57 (45.6)
	***p*-value**	**0.056**
Job	Medical associated	12 (60.0)
	Non-medical	145 (38.2)
	***p*-value**	**0.061**
Nationality	Iranian	154 (40.0)
	Others	3 (20.0)
	***p*-value**	**0.177**
Area of residence	Urban- Capital area	65 (35.9)
	Urban- Provincial area	9 (42.6)
	Rural	83 (41.9)
	***p*-value**	**0.46**
History of MI	Respondent	6 (46.2)
	Immediate family member	47 (33.8)
	None	104 (41.9)
	***p*-value**	**0.255**
Medical Status	Patient with CVD (outpatient/inpatient)	17 (39.5)
	Patient with non-CVD (outpatient/inpatient)	50 (33.1)
	Immediate relative of patients with CVD	20 (45.5)
	Immediate relative of patients with non-CVD	62 (45.9)
	None	8 (29.6)
	***P*-value**	**0.154**
HTN	Yes	17 (27.4%)
	No	140 (41.4%)
	***P*-value**	**.047**
DM	Yes	15 (34.1%)
	No	142 (39.9%)
	***P*-value**	**.515**
HLP	Yes	25 (41.7%)
	No	132 (38.8%)
	***P*-value**	**.670**
IHD	Yes	16 (34.0%)
	No	141 (39.9%)
	***P*-value**	**.525**

*Abbreviations*: *MI* Myocardial Infarction, *CVD* Cardiovascular diseases, *HTN* Hypertension, *DM* Diabetes Mellitus, *HLP* Hyperlipidemia, *IHD* Ischemic Heart Disease

### Source of heath-related information

As shown in Table 7, multiple sources provided health-related data to the study participants. Most respondents reported that television, 186(46.5%), and physicians, 167(41.8%), were their primary sources of health information. Moreover, 36 percent of participants (144 subjects) received health information from social media other than television.

**Table 7:** Sources of health information

Source	Frequency	Percentage
Television	186	46.5
Radio	30	7.5
Friends/family/members/others	159	39.8
Physicians	167	41.8
Social media	144	36.0
web	130	32.5
Magazine	28	7.0
Medical books	61	15.3
Medical staff	32	8.0
Other	37	9.3

## Discussion

This study is the first in Iran to create and evaluate a questionnaire to assess the lay public's understanding and responses to heart attack symptoms. It can be regarded as a valid instrument for future research to determine the lay public's awareness and responses to heart attack symptoms. As patients frequently call a relative following the onset of AMI symptoms, we specifically explored relatives' knowledge and attitudes regarding AMI symptoms in this study. This study population was relatively young, and the majority were residents of urban areas. Although most participants were literate, they needed a better understanding of AMI symptoms.

The current findings would represent the first step in quantifying knowledge of heart attacks and determining the knowledge gaps. This will help assess the adequacy of existing community education programs and could be used to plan future public health promotion efforts. This may help to improve public awareness in the hope of increasing early detection of AMI and reducing the time to treatment.

Previous research has shown that patients may delay seeking treatment for AMI because they believe their symptoms are not cardiac in nature[9, 19]. This issue is more pronounced in patients who feel that only chest pain and not atypical symptoms may be a clinical presentation of the myocardial infarction[20]. In our study, about 62% of participants did not consider neck pain an AMI symptom, consistent with previous studies in Korea and China[13, 16]. Following myocardial infarction, these patients would likely arrive at the hospital with more than 90 min delay, which does not meet the American College of Cardiology/ American Heart Association and European guidelines recommendations[21, 22]. The most common AMI symptom cited by the participants was “chest pain or discomfort,” 285(71.3%), and the last one was “abdominal pain or discomfort,” 56(14.0%). In a study conducted in Poland, 90% of respondents were aware of the symptoms of chest pain, and 27% were aware of the symptoms of arm or shoulder pain[23]; in a study conducted in the United States, 92% of respondents were aware of the symptoms of chest pain and 49.3% were aware of the symptoms of jaw, neck, or back pain[24].

This study observed poor knowledge of AMI symptoms, particularly among people with lower levels of education and those living in provincial or rural areas. We also found that men were less knowledgeable about AMI than women. Inconsistent results have been published regarding gender disparities in AMI knowledge, with some studies indicating that women[10] or men have inferior knowledge[12, 24] and others showing no difference[25, 26].

The frequency of ambulance use was high in response to hypothetical case scenarios in this study, except in the absence of typical chest pain. These findings are consistent with global studies of patients with AMI [27]. In our study, the lack of insight into the critical situation of the patient experiencing MI may be due to unawareness of atypical MI symptoms such as pain in the arm, shoulder, or abdomen. In addition, people may believe they should have more symptoms to call the emergency number or may be afraid to call [28].

In this study, the proportion of respondents with a history of AMI who had excellent knowledge of myocardial infarction symptoms (only one subject (7.7%)) was lower than in the United States [24]. In addition, patients with a history of IHD in our study had a lower awareness of AMI symptoms compared with patients or family members of patients with a history of MI. The perception of severe symptoms in AMI and better patient education by physicians and medical staff may explain this difference. More significant public health efforts should be targeted at patients with ischemic heart disease to increase their awareness of the symptoms of AMI and to emphasize the seriousness of the condition and the urgency of seeking treatment. The crucial role of physicians in raising awareness must be noted, as they were reported to be the primary source of health information for many study participants.

There are notable findings in our study that should be highlighted. The analysis of attributable risk factors showed that study participants considered tobacco/ hookah use, unhealthy diet, insufficient physical activity, obesity, and anxiety as factors associated with AMI. Surprisingly, high LDL levels and diabetes, among the most important risk factors for coronary heart disease, were less recognized as risk factors by the respondents in our study. In contrast, the leading risk factor identified by participants in this survey was anxiety, which is not adequately addressed in the guidelines [29]. Organized community and patient education appear to be essential in this area. Future research should examine awareness of early symptoms in populations at high risk for myocardial infarction (e.g., those with pre-existing coronary artery disease and conditions such as hypertension, diabetes, or dyslipidemia).

Limitations.

This study faced some limitations. First, the information was gathered through a questionnaire and closed-ended questions, which may be associated with an overestimation of the knowledge level of this population. Second, due to the relatively small sample size, future research should provide a more accurate estimate of the population's generalizable community knowledge.

## Conclusion

This is the first study to investigate the knowledge and response of the general public to the symptoms of AMI in Iran. Due to its validity, the questionnaire designed for this study can be effectively used in future studies. To increase awareness of AMI symptoms among the general public in Iran, education and promotion must consider television, social media, and healthcare staff, as these are the primary sources of health information among people. Although there is a need to educate the general population about the symptoms of AMI, special attention should be given to individuals at the highest risk of experiencing an AMI episode. This is a topic that may be worth investigating in the future.

## Data Availability

All data and the questionnaire are available upon request to the corresponding author.

## References

[1] Virani SS, Alonso A, Aparicio HJ, Benjamin EJ, Bittencourt MS, Callaway CW (2021). Heart disease and stroke statistics—2021 update: a report from the American Heart Association. Circulation.

[2] National Heart Lung and Blood Institute. Heart attack, 2017 [Available from: https://www.nhlbi.nih.gov/health/heart-attack.

[3] Vos T, Lim SS, Abbafati C, et al. Global burden of 369 diseases and injuries in 204 countries and territories, 1990–2019: a systematic analysis for the Global Burden of Disease Study 2019. Lancet. 2020;396(10258):1204–22. 10.1016/S0140-6736(20)30925-9 PMC7567026 33069326

[4] Lu L, Liu M, Sun R, Zheng Y, Zhang P (2015). Myocardial infarction: symptoms and treatments. Cell Biochem Biophys.

[5] Gibson CM, de Lemos JA, Antman EM (2004). Time is muscle in primary PCI: the strength of the evidence grows. Eur Heart J.

[6] Goldberg RJ, Spencer FA, Fox KA, Brieger D, Steg PG, Gurfinkel E (2009). Prehospital Delay in Patients With Acute Coronary Syndromes (from the Global Registry of Acute Coronary Events [GRACE]). Am J Cardiol.

[7] Beza L, Leslie SL, Alemayehu B, Gary R (2021). Acute coronary syndrome treatment delay in low to middle-income countries: a systematic review. IJC Heart Vasc.

[8] Mumford AD, Banning AP (1997). Minimising delays to thrombolysis in patients developing acute myocardial infarction in hospital. Postgrad Med J.

[9] Farshidi H, Rahimi S, Abdi A, Salehi S, Madani A (2013). Factors associated with pre-hospital delay in patients with acute myocardial infarction. Iran Red Crescent Med J.

[10] Abed MA, Ali RM, Abu Ras MM, Hamdallah FO, Khalil AA, Moser DK (2015). Symptoms of acute myocardial infarction: A correlational study of the discrepancy between patients' expectations and experiences. Int J Nurs Stud.

[11] Thuresson M, Jarlöv MB, Lindahl B, Svensson L, Zedigh C, Herlitz J (2007). Thoughts, actions, and factors associated with prehospital delay in patients with acute coronary syndrome. Heart Lung.

[12] Ahmed AAA, Al-Shami AM, Jamshed S, Fata Nahas AR (2019). Development of questionnaire on awareness and action towards symptoms and risk factors of heart attack and stroke among a Malaysian population. BMC Public Health.

[13] Park KS (2020). Factors affecting awareness of myocardial infarction symptoms among the general public in Korea. Epidemiol Health.

[14] Henriksson C, Larsson M, Arnetz J, Berglin-Jarlöv M, Herlitz J, Karlsson JE (2011). Knowledge and attitudes toward seeking medical care for AMI-symptoms. Int J Cardiol.

[15] Awad A, Al-Nafisi H (2014). Public knowledge of cardiovascular disease and its risk factors in Kuwait: a cross-sectional survey. BMC Public Health.

[16] Chau PH, Moe G, Lee SY, Woo J, Leung AYM, Chow CM (2018). Low level of knowledge of heart attack symptoms and inappropriate anticipated treatment-seeking behaviour among older Chinese: a cross-sectional survey. J Epidemiol Community Health.

[17] Birnbach B, Höpner J, Mikolajczyk R (2020). Cardiac symptom attribution and knowledge of the symptoms of acute myocardial infarction: a systematic review. BMC Cardiovasc Disord.

[18] Rattray J, Jones MC (2007). Essential elements of questionnaire design and development. J Clin Nurs.

[19] Leslie WS, Urie A, Hooper J, Morrison CE (2000). Delay in calling for help during myocardial infarction: reasons for the delay and subsequent pattern of accessing care. Heart.

[20] Horne R, James D, Petrie K, Weinman J, Vincent R (2000). Patients' interpretation of symptoms as a cause of delay in reaching hospital during acute myocardial infarction. Heart.

[21] Ibanez B, James S, Agewall S, et al. 2017 ESC Guidelines for the management of acute myocardial infarction in patients presenting with ST-segment elevation: the Task Force for the management of acute myocardial infarction in patients presenting with ST-segment elevation of the European Society of Cardiology (ESC). Eur Heart J. 2018;39(2):119–77. 10.1093/eurheartj/ehx393 28886621

[22] Levine GN, Bates ER, Blankenship JC, et al. 2015 ACC/AHA/SCAI focused update on primary percutaneous coronary intervention for patients with ST-elevation myocardial infarction: an update of the 2011 ACCF/AHA/SCAI guideline for percutaneous coronary intervention and the 2013 ACCF/AHA guideline for the management of ST-elevation myocardial infarction. Circulation. 2016;133(11):1135–47. 10.1161/CIR.0000000000000336 26490017

[23] Kopec G, Sobien B, Podolec M, Dziedzic H, Zarzecka J, Loster B (2011). Knowledge of a patient-dependant phase of acute myocardial infarction in Polish adults: the role of physician's advice. Eur J Public Health.

[24] Fang J, Gillespie C, Keenan NL, Greenlund KJ (2011). Awareness of heart attack symptoms among US adults in 2007, and changes in awareness from 2001 to 2007. Future Cardiol.

[25] Ratner PA, Tzianetas R, Tu AW, Johnson JL, Mackay M, Buller CE (2006). Myocardial infarction symptom recognition by the lay public: the role of gender and ethnicity. J Epidemiol Community Health.

[26] Ek S (2015). Gender differences in health information behaviour: a Finnish population-based survey. Health Promot Int.

[27] Mujtaba SF, Sohail H, Ram J, Waqas M, Hassan M, Sial JA (2021). Pre-hospital delay and its reasons in patients with acute myocardial infarction presenting to a primary percutaneous coronary intervention-capable center. Cureus.

[28] Lozzi L, Carstensen S, Rasmussen H, Nelson G (2005). Why do acute myocardial infarction patients not call an ambulance? An interview with patients presenting to hospital with acute myocardial infarction symptoms. Intern Med J.

[29] Celano CM, Millstein RA, Bedoya CA, Healy BC, Roest AM, Huffman JC (2015). Association between anxiety and mortality in patients with coronary artery disease: a meta-analysis. Am Heart J.

